# Evaluation of central venous stenosis using the lateral thoracic pathway on low-dose CT

**DOI:** 10.3389/fmed.2026.1727004

**Published:** 2026-06-19

**Authors:** Guo Li, Ge Zhang, Xiaoyi Wang, Shishi Luo, Xin Qin, Shanxi Guo, Wangsheng Chen, Weiyuan Huang, Feng Chen

**Affiliations:** 1 Hainan General Hospital, Haikou, China; 2 Hainan Affiliated Hospital of Hainan Medical University, Haikou, China

**Keywords:** central venous stenosis, collateral circulation, hemodialysis, lateral thoracic pathway, low-dose computed tomography

## Abstract

**Objective:**

Collateral circulation is a biomarker of central venous stenosis (CVS). We attempted to identify the presence of CVS in hemodialysis patients using the lateral thoracic pathway (LTP) on low-dose computed tomography (CT).

**Methods:**

A total of 355 patients with end-stage renal disease were retrospectively enrolled in this study and further divided into three subgroups (central vein catheters, CVC, 121; arteriovenous fistulas, AVF, 162; and arteriovenous grafts, AVG, 72). All patients underwent low-dose CT within 10 days prior to the intervention, and CT venography was used for demonstration if it was performed during this period. Patients were defined as positive LTP when vascular-like attenuation was present on either side of the anterolateral chest wall. The incidence of thoracic varices from physical examination and LTP on low-dose CT was calculated, and their sensitivity, specificity, positive predictive value, and negative predictive value for differentiating CVS were analyzed.

**Results:**

Compared with the non-CVS group, comorbidities were more common in the CVS group, and the access age was also longer (all *P* < 0.05). The rate of positive LTP is higher than that of thoracic varices (54.4% vs. 12.1%). There was no statistically significant difference in age and sex between the CVS and non-CVS (all *P* > 0.05), but thoracic varices and positive LTP were significantly higher in the CVS than in the non-CVS (all *P* < 0.001). In all groups, the sensitivity of LTP was significantly higher than that of the thoracic varices (CVC:0.939 vs. 0.303; AVF:0.956 vs. 0.400; AVG:0.917 vs. 0.208) and maintained a high negative predictive value (CVC:0.966; AVF:0.974; AVG:0.926). The Cohen’s kappa values showed excellent reproducibility between the two radiologists (κ = 0.977).

**Conclusion:**

Negative LTP help identify hemodialysis patients at lower risk of CVS and may reduce unnecessary interventions.

## 1 Introduction

Central venous stenosis (CVS) is a common issue in vascular access (1). It was estimated that CVS occurs in 25–40% of hemodialysis patients (2). Stenosis or occlusion of major intrathoracic veins can lead to ineffective dialysis and progressive venous hypertension (3). In the past, intervention has been used as the gold standard for assessing stenosis, as well as a therapeutic solution (1, 3). However, this is an invasive procedure and also unnecessary for most patients. Moreover, central venous imaging is commonly performed during peripheral dialysis access interventions. Additional ionizing radiation and contrast application are inevitable in this case.

The clinical signs (e.g., limb swelling) and symptoms (e.g., thoracic varices) of CVS have been explored in previous studies (4, 5). Unfortunately, < 50% of these patients had positive results. Subsequently, noninvasive vascular imaging was favored and developed (6–8). Ultrasound via the supraclavicular route allows for assessment of the subclavian and brachiocephalic veins, but visualization of the superior vena cava is poor (9). Non-contrast-enhanced MR venography remains an expensive and relatively limited-access technique (10). Cross-sectional CT venography provides the best answer to date. Notably, fistulas and graft-induced hemodynamic changes leading to higher radiation persist (7, 8). Meanwhile, contrast injection can cause further damage to renal function. Even with excellent imaging, there are still some challenges in vascular assessment. Deformed normal veins are often incorrectly interpreted as venous stenosis. Central venous catheter also affects the visualization of veins due to fibrin sheaths (11).

When CVS is present, the obstructed flow will be compensated through the collateral veins (12). They are usually categorized into four main collateral pathways, such as the lateral thoracic, internal mammary, azygos, and vertebral pathways (13). Most veins of the lateral thoracic pathway (LTP), such as lateral thoracic vein and thoracoepigastric vein, ascend superficially on the anterolateral chest and abdominal wall. These veins are easily visualized by CT owing to the attenuation of fatty tissue. Conversely, other pathways located within the thoracic cavity are not easily recognized by unenhanced CT due to similar attenuation. It may be a quick and easy way to identify CVS by characterizing LTP on unenhanced CT. Opportunely, low-dose CT is routinely used to evaluate heart and lung disease (14). Not only that, additional benefits have been confirmed by recent studies (15, 16). Based on the above understanding, we attempted to evaluate whether LTP on low-dose CT could identify the presence of CVS.

## 2 Materials and methods

### 2.1 Study patients

**FIGURE 1:**
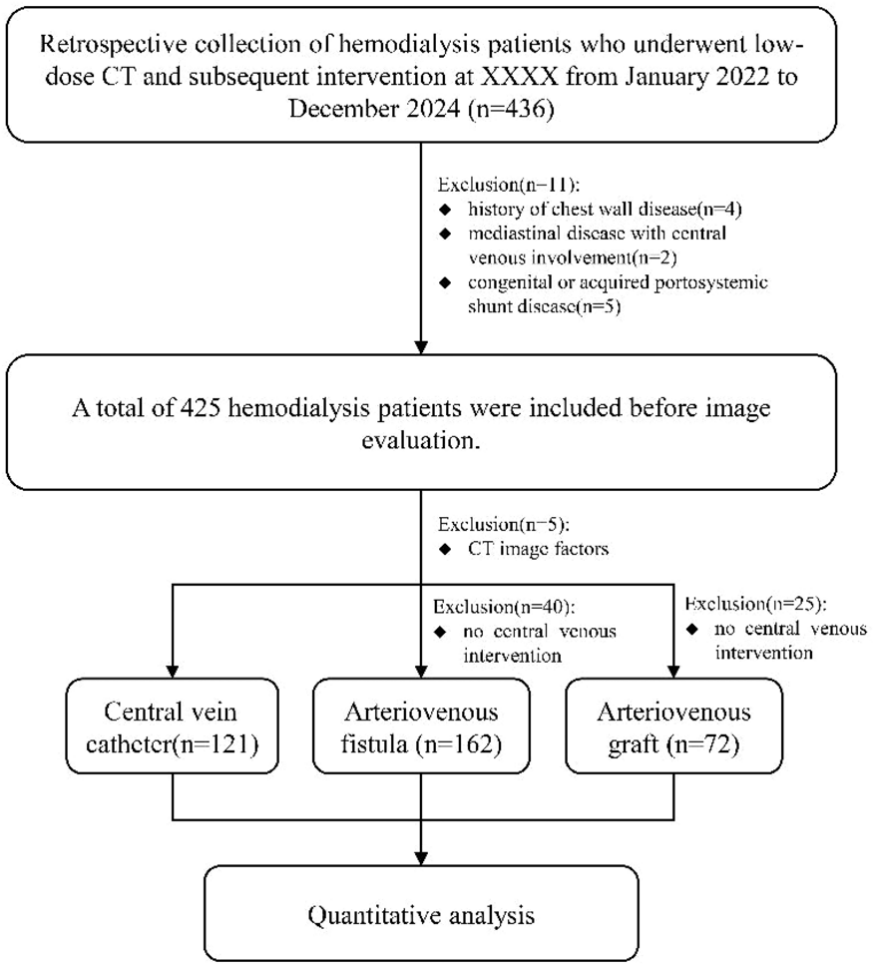
Outline of the study protocol.

The research protocol was approved by the Institutional Review Committee of the Academy. An investigator retrospectively searched for hemodialysis patients with electronic medical records archived from January 2022 to December 2024 at XXXX. Patients were included in this study if they underwent a low-dose CT scan and received interventional treatment within 10 days of the CT scan. If the patient performed CT venography (CTV) during this period, the CTV was used for solely for illustrative purposes in this study. Low-dose CT was conducted for evaluating cardiopulmonary disease, and CTV was performed to identify the presence of CVS. Exclusion criteria: (1) history of chest wall disease (e.g., trauma, infection, hematoma, tumor, congenital injury, etc.); (2) mediastinal disease with central vein involvement, e.g., tumor, infection, hematoma; (3) congenital or acquired portosystemic shunt disease such as significant portal hypertension due to liver cirrhosis; (4) CT image factors, such as artifacts, that interfere with chest wall assessment; (5) central vein was not evaluated during the intervention. For example, in some patients with AVF or AVG, only the fistula and graft were assessed during the interventional procedure. Sex, age, and clinical signs or symptoms were recorded for all patients. According to the hemodialysis route, the patients were further divided into the following subgroups: central vein catheter group (Group A), arteriovenous fistula group (Group B), and arteriovenous graft group (Group C). The selection of patients for this study is shown in Figure 1.

### 2.2 CT protocol

All low-dose CT scans were compliant with the International Early Lung Cancer Action Plan (I-ELCAP) protocol (17). Volumetric CT were performed using a multidetector CT scanner (Philips Healthcare, Siemens Healthcare, or General Electric Healthcare) without contrast administration. Low-dose CT scans were designed according to the following standardized protocol: 120 kVp tube voltage, 30 mAs tube current (automatic tube current adjustment disabled), 0.625 mm or equivalent collimator, 0.5 s rotation time, 1.375 pitch, and 0.5 mm or 0.625 mm slice thickness. All images were reconstructed using standardized reconstruction kernels: each vendor employed a sharp reconstruction kernel for the lungs (e.g., Philips “Lung,” Siemens “B70f,” GE “Lung”) and a soft-tissue reconstruction kernel for the mediastinum (e.g., Philips “Standard,” Siemens “B31f,” GE “Standard”). The reconstruction algorithms used were model-based iterative reconstruction methods. In addition, 3.0-mm images in the sagittal and coronal planes were also acquired. This is because reconstructed images aid in the diagnosis of LTP. The Philips, Siemens, and GE scanners were used to perform low-dose CT on 77, 184, and 94 patients, respectively. The CT dose index volume (CTDIvol) and dose length product (DLP) were extracted directly from each patient’s dose report. The actual radiation dose was 1.83 ± 0.31 mGy (CTDIvol) and 52.5 ± 8.6 mGy⋅cm (DLP), corresponding to an effective dose of approximately 0.71 ± 0.13 mSv.

### 2.3 The LTP

**FIGURE 2:**
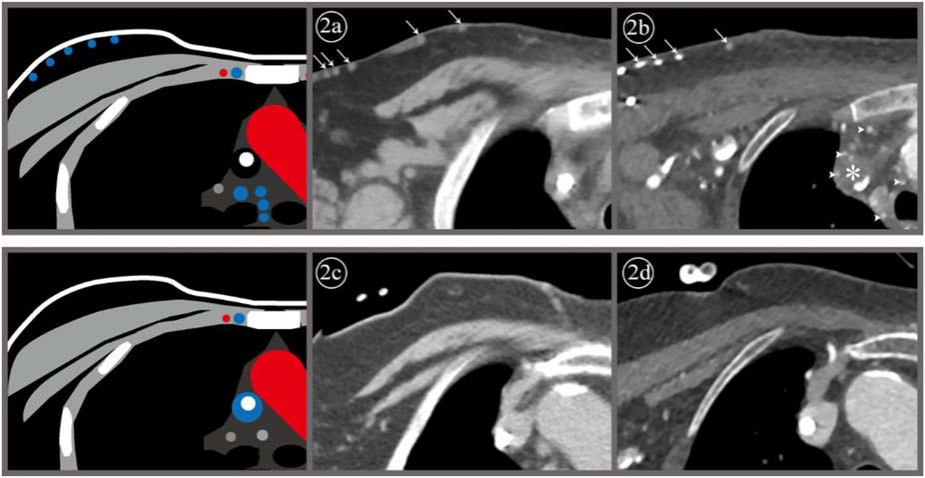
Positive LTP: Vascular-like attenuation was observed in the anterolateral chest wall on low-dose CT (**a**, arrows). The vascular-like attenuation shown by low-dose CT was confirmed in the CT venography (**b**, arrows). Note the protein sheath around the central venous catheter resulting in stenosis of the superior vena cava (asterisks), along with multiple collateral veins developing in the mediastinum (arrowheads). Negative LTP: No vascular-like attenuation of the anterolateral chest wall was present on low-dose CT and CT venography **(c,d)**.

The exact definition of the LTP is as follows: attenuation of vascular-like tissue on the anterolateral chest wall and/or abdominal wall (Figure 2). Additionally, this sign could be categorized as unilateral and bilateral. Positive LTP were defined when the sign appeared on either side; conversely, they were defined as negative. If more than one examination was performed prior to the intervention, the patient was defined as a positive LTP patient once signs were observed. The window level for image interpretation is 50–150 HU and the window width is 120–300 HU. All images were independently characterized by two radiologists (with more than 8 years of experience) and discrepancies were resolved by consensus by a third radiologist (with more than 10 years of experience). All radiologists evaluated only low-dose CT, excluding clinical history, CTV images and interventional outcomes. The images interpreted by the two radiologists were randomly selected by a senior radiologist. Since the LTP is not complicated, the initial assessment was completed within a week. The above evaluation was repeated 2 weeks later. The discrepancies between each radiologist’s results were resolved through consultation with a senior radiologist.

### 2.4 Intervention

Interventional images were stored in digital format in Picture Archiving and Communication Systems (PACS) to ensure that all images of patients in this study were available for diagnostic purposes. All diagnoses were performed independently by an interventionalist with 15 years of experience in endovascular dialysis access procedures. The interventionalist was only informed of the patient’s sex, age, and history of hemodialysis, and was not aware of the patient’s CT findings or endovascular treatment. According to the guidelines, the superior vena cava, brachiocephalic vein and subclavian vein constitute the central vein (1). Any venous stenosis > 25% was diagnosed as CVS, while stenosis < 25% was classified as non-CVS. Patients with CVS were further divided into symptomatic and asymptomatic according to whether the stenosis was more than 50% (18). Symptomatic CVS was defined as a stenosis of more than 50%, while asymptomatic CVS was defined as a stenosis of < 50%. Symptomatic patients underwent balloon angioplasty and intraluminal stenting as necessary. Asymptomatic CVS and non-CVS patients were followed up only without any treatment.

### 2.5 Statistical analysis

Continuous data were expressed as mean ± standard deviation or median and interquartile range. Categorical data were expressed as counts. After assessment of normality using the Shapiro-Wilk test, continuous data were analyzed with the independent samples *t*-test or the Mann-Whitney U test. Categorical data were compared using the chi-square test. McNemar test and Cohen’s kappa were used to assess inter-observer agreement. All analyses were performed using Statistical Package for the Social Sciences (SPSS) software (version 22.0, IBM). Differences were considered statistically significant at *P* < 0.05.

## 3 Results

### 3.1 Characteristics

Of the 436 hemodialysis patients, 355 met the inclusion criteria, and 81 were excluded because of a history of chest wall disease (2 cases with breast cancer, 1 case with breast cancer surgery, 1 case with trauma), mediastinal lesion involving the central vein (1 case with lung cancer, 1 case with mediastinitis), significant portal hypertension due to liver cirrhosis (4 cases), abernethy malformation (1 case), imaging factors (5 cases), or lack of central venous intervention (65 cases). The demographic characteristics of hemodialysis patients are shown in Table 1. There were no significant differences between the CVS and non-CVS groups in age (*P* = 0.179) and sex (*P* = 0.503). Comorbidities were more common in CVS than in non-CVS (all *P* < 0.05). Swelling, especially asymmetric swelling, was more common in CVS (all *P* < 0.05). The incidence of thoracic varices was significantly higher in the CVS than in the non-CVS (32.4% vs. 4%). The classification of vascular access was not significantly different between the two groups (*P* > 0.05), but the age of vascular access was significantly higher in CVS than in non-CVS (6.3 ± 3.8y vs. 3.6 ± 3.5y). Positive LTP was more common in the CVS group (94.1% vs. 38.3%).

**TABLE 1:** Characteristics of hemodialysis patients.

Variable	CVS (*n* = 102)	Non-CVS (*n* = 253)	*P*
Sex			0.503
Male	48 (47.1%)	129 (51.0%)	
Female	54 (52.9%)	124 (49.0%)	
Age (years)	60.1 ± 14.4	57.1 ± 13.5	0.179
Comorbidities			
Diabetes	55 (53.9%)	79 (31.2%)	< 0.001
PAD	33 (32.4%)	47 (18.6%)	0.005
CVD	62 (60.8%)	51 (20.2%)	< 0.001
Clinical symptoms			
Swelling	41 (40.2%)	68 (26.9%)	0.014
Asymmetry	27 (26.5%)	22 (8.7%)	< 0.001
Symmetry	14 (13.7%)	46 (18.2%)	0.311
Cutaneous findings			
Skin discoloration	20 (19.6%)	32 (12.6%)	0.093
Thoracic varices	33 (32.4%)	10 (4.0%)	< 0.001
Phlebitis	3 (2.9%)	9 (3.6%)	1.000
Persistent pain	25 (24.5%)	37 (14.6%)	0.026
Vascular access			
CVC	33 (32.4%)	88 (34.8%)	0.662
AVF	45 (44.1%)	117 (46.2%)	0.716
AVG	24 (23.5%)	48 (19.0%)	0.334
Access age (years)	6.3 ± 3.8	3.6 ± 3.5	< 0.001
Positive-LTP	96 (94.1%)	97 (38.3%)	< 0.001
Lesions			
Sites			
Subclavian vein	18 (17.6%)	-	
Brachiocephalic vein	45 (44.1%)	-	
Superior vena cava	39 (38.3%)	-	
Intervention			
Angioplasty	87 (85.3%)	-	
Stent	7 (6.9%)	-	
Balloon size			
Diameter (mm)			
Mean	9.75	-	
Range	8–12	-	
Length (mm)			
Mean	60	-	
Range	40–80	-	

CVS, central venous stenosis; PAD, peripheral arterial disease; CVD, cardiovascular disease; CVC, central venous catheters; AVF, arteriovenous fistula; AVG, arteriovenous graft; LTP, lateral thoracic pathways.

### 3.2 Diagnostic performance of thoracic varices and LTP

**FIGURE 3:**
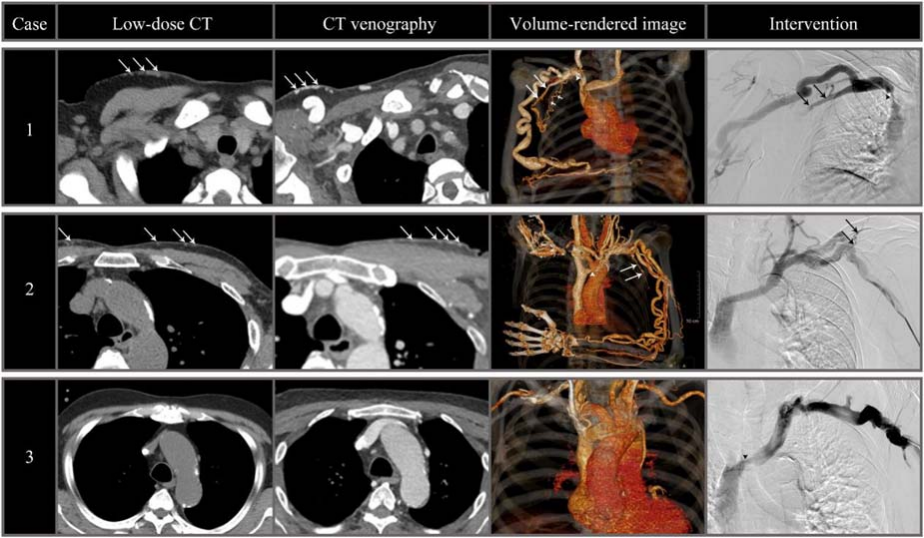
Low-dose CT, CT venography and corresponding interventional images in hemodialysis patients. Case 1. 57y, male, positive thoracic varices. Positive LTP of the right anterolateral chest wall was demonstrated on low-dose CT (arrows), which was also confirmed on subsequent CT venography (arrows). Note the difference in LTP on CT scans due to arm position. The volume-rendered image showed a marked stenosis of the right subclavian vein (arrowhead), while significant dilatation of the LTP (long arrow) and the internal mammary vein (short arrow) could also be observed. Intervention confirmed the site of stenosis (black arrowhead) as well as activated LTP (black arrow). Case 2. 63y, male, negative thoracic varices. Both low-dose CT (arrows) and CT venography (arrows) revealed positive LTP. The volume-rendered image showed a marked stenosis of the left brachiocephalic vein (arrowhead) and a positive LTP (arrows). No central venous stenosis was detected by intervention, but positive LTP was indicated (black arrow). Case 3. 62y, male, negative thoracic varices. Low-dose CT and CT venography suggested negative LTP, and central venous stenosis and collateral pathways were not detected on volume-rendered images. Intervention indicated mild stenosis of the left brachiocephalic vein (black arrowhead).

Interventional results are listed in Table 2, and the predictive value of thoracic varices and LTP for CVS was also assessed. The incidence of positive LTP was higher than that of thoracic varices in groups A, B and C. The specificity of thoracic varices in predicting CVS was high, ranging from 0.940 to 0.979. However, the sensitivity is relatively low, at 0.400 or less. The sensitivity of LTP to predict CVS was good, ranging from 0.917 to 0.956. The sensitivity was also as high as 0.941 in all patients. Although the specificity was relatively low (0.617), there was a high negative predictive value (0.963) in all patients (Figure 3). There was no significant difference in the rate of positive LTP among non-CVS patients across the groups (*P* = 0.309).

**TABLE 2:** Diagnostic performance of thoracic varices and LTP in differentiating CVS.

Variable	Status	CVS,n (%)		*P*	Sensitivity (95%CI)	Specificity (95%CI)	PPV (95%CI)	NPV (95%CI)
		+	-					
Group A								
Thoracic varices	+	10 (30.3%)	2 (2.3%)	< 0.001	0.303 (0.173–0.468	0.977 (0.921–0.995)	0.833 (0.554–0.953)	0.789 (0.703–0.855)
	−	23 (69.7%)	86 (97.7%)					
Unilateral LTP	+	28 (84.8%)	16 (18.2%)	< 0.001	0.848 (0.688–0.938)	0.818 (0.723–0.888)	0.636 (0.486–0.766)	0.935 (0.856–0.973)
	−	5 (15.2%)	72 (81.8%)					
Bilateral LTP	+	3 (9.1%)	15 (17.0%)	0.274	0.091 (0.031–0.241)	0.830 (0.736–0.897)	0.167 (0.058–0.394)	0.709 (0.614–0.789)
	−	30 (90.9%)	73 (83.0%)					
Unilateral or bilateral LTP	+	31 (93.9%)	31 (35.2%)	< 0.001	0.939 (0.801–0.984)	0.648 (0.543–0.741)	0.500 (0.378–0.622)	0.966 (0.883–0.992)
	−	2 (6.1%)	57 (64.8%)					
Group B								
Thoracic varices	+	18 (40.0%)	7 (6.0%)	< 0.001	0.400 (0.265–0.550)	0.940 (0.879–0.973)	0.720 (0.519–0.861)	0.803 (0.725–0.864)
	−	27 (60.0%)	110 (94.0%)					
Unilateral LTP	+	33 (73.3%)	36 (30.8%)	< 0.001	0.733 (0.587–0.844)	0.692 (0.603–0.770)	0.478 (0.362–0.597)	0.871 (0.787–0.926)
	−	12 (26.7%)	81 (69.2%)					
Bilateral LTP	+	10 (22.2%)	7 (6.0%)	0.002	0.222 (0.125–0.362)	0.940 (0.879–0.973)	0.588 (0.360–0.788)	0.759 (0.683–0.822)
	−	35 (77.8)	110 (94.0%)					
Unilateral or bilateral LTP	+	43 (95.6%)	43 (36.8%)	< 0.001	0.956 (0.845–0.989)	0.632 (0.542–0.714)	0.500 (0.396–0.604)	0.974 (0.910–0.994)
	−	2 (4.4%)	74 (63.2%)					
Group C								
Thoracic varices	+	5 (20.8%)	1 (2.1%)	0.003	0.208 (0.092–0.406)	0.979 (0.886–0.997)	0.833 (0.435–0.975)	0.712 (0.595–0.806)
	−	19 (79.2%)	47 (97.9%)					
Unilateral LTP	+	15 (62.5%)	15 (31.2%)	0.011	0.625 (0.424–0.791)	0.688 (0.545–0.802)	0.500 (0.330–0.670)	0.786 (0.637–0.886)
	−	9 (37.5%)	33 (68.8%)					
Bilateral LTP	+	7 (29.2%)	8 (16.7%)	0.218	0.292 (0.148–0.484)	0.833 (0.698–0.918)	0.467 (0.243–0.706)	0.702 (0.572–0.805)
	−	17 (70.8%)	40 (83.3%)					
Unilateral or bilateral LTP	+	22 (91.7%)	23 (47.9%)	< 0.001	0.917 (0.736–0.979)	0.521 (0.380–0.659)	0.489 (0.346–0.634)	0.926 (0.761–0.983)
	−	2 (8.3%)	25 (52.1%)					
Total								
Thoracic varices	+	33 (32.4%)	10 (4.0%)	< 0.001	0.324 (0.244–0.413)	0.960 (0.926–0.980)	0.767 (0.621–0.870)	0.779 (0.730–0.822)
	−	69 (67.6%)	243 (96.0%)					
Unilateral LTP	+	76 (74.5%)	67 (26.5%)	< 0.001	0.745 (0.653–0.821)	0.735 (0.677–0.787)	0.531 (0.449–0.612)	0.877 (0.826–0.916)
	−	26 (25.5%)	186 (73.5%)					
Bilateral LTP	+	20 (19.6%)	30 (11.9%)	0.057	0.196 (0.130–0.285)	0.881 (0.835–0.917)	0.400 (0.274–0.540)	0.731 (0.679–0.778)
	−	82 (80.4%)	223 (88.1%)					
Unilateral or bilateral LTP	+	96 (94.1%)	97 (38.3%)	< 0.001	0.941 (0.876–0.974)	0.617 (0.553–0.677)	0.497 (0.427–0.568)	0.963 (0.923–0.983)
	−	6 (5.9%)	156 (61.7%)					

LTP, lateral thoracic pathways; CVS, central venous stenosis; CI, confidence interval; PPV, positive predictive value; NPV, negative predictive value.

### 3.3 Reproducibility of LTP

McNemar test showed that there was no significant difference in image interpretation between the two radiologists (*P* = 0.134) (Table 3). The kappa coefficient was 0.977, indicating excellent agreement in image interpretation between the two observers.

**TABLE 3:** Interobserver reliability for the LTP.

Variable	Status	Radiologist1	Radiologist2	*P* (McNemar test)	κ
LTP	+	192	196	0.134	0.977
	−	163	159		

LTP, lateral thoracic pathways.

## 4 Discussion

In clinical practice, we have noted that LTP due to CVS can be easily detected with naked eye on CT even without contrast injection. Associated with lung nodule follow-up and COVID-19 pandemic, low-dose CT is a routine tool in daily work (19, 20). We conducted this study and found that LTP is common in hemodialysis patients (193 of 355). Meanwhile, LTP had a very high sensitivity (0.941) and negative predictive value (0.963) for identifying CVS.

Considering the possible impact of different vascular access on CVS, all patients were further divided into three subgroups. In each subgroup, positive LTP was as high as about 50% or more. Not only that, LTP showed high sensitivity in all three subgroups (0.917–0.956). However, its specificity is relatively low (0.521–0.648). The low specificity may be due to hemodynamic changes caused by arteriovenous fistula or graft (3). Both arterial and venous hemodynamics are altered after access is created, leading to an increase in vascular diameter, blood flow, and blood volume within the circulation. Circulatory burden can be further increased by repetitive insertions, graft and vein injuries (2). It is not surprising that collateral vein pathways, such as LTP, are activated as compensatory routes even in the absence of CVS (21). Central venous catheter as an adjunct when arteriovenous fistula or graft is not available (22). The effects of hemodynamic changes remain. In addition, LTP calcification may be misdiagnosed as positive (3 of 355). We also observed 6 cases of missed CVS in this study (two in each group). These CVS were located in the superior vena cava (2 cases) and the brachiocephalic vein (4 cases). All of these patients had asymptomatic CVS not requiring endovascular treatment. This also means that intervention is not necessary in these patients. Furthermore, LTP was not detected during the intervention.

As previously described, CVS can be compensated via four collateral pathways (13). These pathways are not independent, but relieve obstruction by interacting with each other (6, 7, 13). When the CVS is located in the subclavian vein, blood flow is reversed into the axillary vein—lateral thoracic vein—LTP and back to the heart through other pathways (Figure 3). Similarly, when the obstruction is located in the brachiocephalic vein or superior vena cava, blood flow is compensated through the internal mammary vein-lateral thoracic vein or the azygos vein- internal mammary vein-lateral thoracic vein. Thus, CVS at either site resulted in activation of the collateral pathway including LTP. These have been illustrated by previous works (13, 21, 23). Also, endovascular therapy uses collateral pathways as a criterion for efficacy (24).

Previous studies have shown that the symptoms or signs are present in < 50% of patients with CVS (4, 5). The results in this study were consistent with their findings. We also evaluated the performance of thoracic varices for predicting CVS. Despite the higher specificity, the sensitivity of the thoracic varices was low, at 20% or less. This can be explained by the size and location of the collateral veins. Smaller diameter veins and distance from the skin are not easily detected by physical examination. Cross-sectional CT images are a feasible solution to this missed diagnosis of LTP. In addition, since the combination of thoracic wall varicose veins and LTP did not significantly improve CVS evaluations, it was not included in the results. Developing a clinical-LTP model may help improve the ability to assess CVS. However, we found that LTP and CVS exhibited complete (or nearly complete) separation. Consequently, the odds ratios obtained from both univariable and multivariable logistic regression were unstable. Therefore, the results of the logistic regression were not included in this paper.

When LTP is used to assess CVS, it still needs to be differentiated from several diseases. Chest wall disorders such as infection or scar have the potential to mimic LTP and lead to misdiagnosis. Tumors that invade the central vein and portosystemic shunt disease can also cause collateral dilatation (25). Another potential factor is arterial collateral due to aortic stenosis. Aortoiliac occlusive disease induces compensation of the systemic circulation (e.g., Winslow pathway) and visceral pathways (e.g., Ryland’s arch) (26). Unlike LTP, these pathways are deep in the chest and abdominal wall and cannot be detected by unenhanced CT (27, 28).

This study still has some limitations. First, selective bias in retrospective studies cannot be avoided. Our findings were based on a population undergoing central venous intervention and still need to be validated in a large cohort of hemodialysis patients. Especially in patients with AVF and AVG, as they may not require central venous intervention. Second, limitations in sample size may affect the analysis of the results. For example, we only included 72 patients in the AVG group. Third, we did not further identify the two types of CVS. Since symptomatic CVS requires endovascular treatment whereas asymptomatic CVS does not. Accurate differentiation can further reduce unnecessary interventions. Insufficient patients with asymptomatic CVS in our study might impact the assessment. Finally, hemodialysis patients with inferior vena cava or femoral vein obstruction were not included in this article. These central veins are not routine dialysis access in our center and appropriate cases are unavailable. Of course, we believe that LTP can still identify CVS given the same mechanism (21, 23). Further validation of the lower central vein could benefit more patients.

In conclusion, assessment of LTP by low-dose CT can be a simple and easy-to-use indicator for characterizing CVS. Negative LTP may serve as a useful rule-out indicator when interpreted together with clinical findings. Positive LTP should be used cautiously for identifying CVS due to its relatively low specificity and modest positive predictive value.

## Data Availability

The raw data supporting the conclusions of this article will be made available by the authors, without undue reservation.
