# Correction: Evaluation of central venous stenosis using the lateral thoracic pathway on low-dose CT

**DOI:** 10.3389/fmed.2026.1931465

**Published:** 2026-07-22

**Authors:** Guo Li, Ge Zhang, Xiaoyi Wang, Shishi Luo, Xin Qin, Shanxi Guo, Wangsheng Chen, Weiyuan Huang, Feng Chen

**Affiliations:** 1 Hainan General Hospital, Haikou, China; 2 Hainan Affiliated Hospital of Hainan Medical University, Haikou, China

**Keywords:** central venous stenosis, collateral circulation, hemodialysis, lateral thoracic pathway, low-dose computed tomography

Author Ge Zhang was erroneously omitted as equal contributing author. The original version of this article has been updated.

